# Broadband Optical Cavity Mode Measurements at Hz-Level Precision With a Comb-Based VIPA Spectrometer

**DOI:** 10.1038/s41598-019-44711-4

**Published:** 2019-06-03

**Authors:** Grzegorz Kowzan, Dominik Charczun, Agata Cygan, Ryszard S. Trawiński, Daniel Lisak, Piotr Masłowski

**Affiliations:** Institute of Physics, Faculty of Physics, Astronomy and Informatics, Nicolaus Copernicus University in Toruń, Ul. Grudziadzka 5, 87-100 Toruń, Poland

**Keywords:** Near-infrared spectroscopy, Atomic and molecular interactions with photons, Near-infrared spectroscopy

## Abstract

Optical frequency comb spectrometers open up new avenues of investigation into molecular structure and dynamics thanks to their accuracy, sensitivity and broadband, high-speed operation. We combine broadband direct frequency comb spectroscopy with a dispersive spectrometer providing single-spectrum acquisition time of a few tens of milliseconds and high spectral resolution. We interleave a few tens of such comb-resolved spectra to obtain profiles of 14-kHz wide cavity resonances and determine their positions with precision of a few hertz. To the best of our knowledge, these are the most precise and highest resolution spectral measurements performed with a broadband spectrometer, either comb-based or non-comb-based. This result pushes the limits of broadband comb-based spectroscopy to Hz-level regime. As a demonstration of these capabilities, we perform simultaneous cavity-enhanced measurements of molecular absorption and dispersion, deriving the gas spectra from cavity mode widths and positions. Such approach is particularly important for gas metrology and was made possible by the Hz-level resolution of the system. The presented method should be especially applicable to monitoring of chemical kinetics in, for example, plasma discharges or measurements of narrow resonances in cold atoms and molecules.

## Introduction

Direct frequency comb spectroscopy (DFCS) employs a light source virtually equivalent to thousands of narrow and highly stable continuous-wave (CW) lasers to simultaneously interrogate multiple atomic or molecular transitions. The high sensitivity, speed and accuracy at which it operates enabled novel experiments in environmental monitoring^1^, high-temperature line-shape parameters retrieval^2^, chemical kinetics at microsecond timescale^3^ and continuous probing of cold complex molecules^4^ among others, while additional applications continue being developed. Frequency metrology applications of optical frequency combs (OFCs) are usually based on heterodyne beat measurements of a single comb line with a CW laser, with signals originating from the other comb lines being filtered out, which makes it relatively easy to take advantage of the ultimate precision and accuracy of stabilized OFCs. A unique challenge in developing DFCS techniques is the need to perform massively parallel detection without inordinately degrading performance in other aspects, especially in frequency resolution or spectral shapes retrieval, which are common in incoherent light-based broadband spectroscopy^5^. Although the potential to fully exploit precision and accuracy of stabilized OFCs by comb-resolving spectroscopic systems was recognized early^6^, the widths of the measured features in top-level demonstrations were limited to hundreds of kilohertz^6–8^. In dual-comb spectroscopy^9–11^ two OFCs with slightly detuned repetition rates are beaten with each other to produce a time-domain signal, which after performing Fourier transform returns the original spectrum. With this approach compact systems offering millisecond-level acquisition are possible at the cost of spectral coverage and either technically difficult mutual synchronization of the OFCs or interferogram post-processing^12,13^. Operating on the same physical basis, mechanical Michelson interferometers offer broadband and sensitive detection with measurement times of a few seconds, limited by the mirror movement speed. In both dual-comb and mechanical systems careful adjustment of the recorded interferogram length was shown to result in resolution beyond the Fourier limit^14,15^, in principle limited only by the frequency stability of the OFC. The limits of the width of the absorption features measured by these systems were set by cavity mode resonances of a few MHz in ref.^16^ and 191 kHz in refs^17,18^. The so-called virtually-imaged phased-array (VIPA) spectrometer, a highly dispersive side-entrance etalon coupled with a perpendicularly-aligned diffraction grating, has been used for broadband spectroscopy with moderately high resolution^3,19^ or for comb-resolved measurements in a reduced spectral range^8,20^. It is uniquely suited to time-resolved experiments, enabling triggered broadband spectral acquisition in time as short as few microseconds, limited by the photodetector array speed.

Cavity-enhanced techniques^21^ provide a way to increase sensitivity of laser spectroscopy, effectively increasing the absorption length by up to five orders of magnitude. In its most straightforward form the sample absorption is determined by directly measuring attenuation of the probe laser by the sample. This technique has been successfully combined with OFCs leading to broadband and highly sensitive measurements^22–25^. In cavity ringdown spectroscopy (CRDS)^26^, sample absorption is derived from decay time constant of the light leaking from the cavity, which shortens with increasing absorption. While the technique effectively removes deleterious effects of intensity noise on the spectra and obviates the need for acquiring reference spectra, its stringent requirements on speed and linearity of the detection system^27^ are at odds with its simultaneous broadband operation, which makes the technique less attractive for combining with OFCs^28^. Its Fourier inverse, cavity mode-width spectroscopy (CMWS)^29^, and cavity mode-dispersion spectroscopy (CMDS)^30^ depend on the ability to precisely measure shapes and positions of narrow cavity modes, trading the requirement of high-speed operation of the detection system for relative frequency stability of the laser and the cavity. While the CMWS technique utilizes broadening of cavity modes to determine sample absorption, CMDS makes use of the fact that cavity modes are shifted by sample dispersion. In the latter case, as measurements are based purely on frequency determination of mode centers, they are less susceptible to systematic errors in intensity measurements caused, for example, by detector nonlinearity.

Recently, an OFC with a Fourier-transform spectrometer was used to measure positions of 191 kHz-wide cavity modes with 20 kHz-wide instrumental function, to characterize dispersion of cavity mirrors and obtain broadband dispersion spectra of CO_2_-N_2_^17^. In this article, we report measurements of 15 times narrower cavity modes with 900 times narrower instrumental function, resulting in few-Hz precision of cavity mode width and position determination and demonstrating the most exact application of the OFC frequency precision in a broadband spectroscopic system. We perform simultaneous absorption and dispersion spectra measurements and confirm the observation from CW laser-based measurements that dispersion measurements offer better precision and accuracy than absorption ones. The careful analysis of molecular lineshapes did not reveal any distortions, verifying that the spectrometer fully resolved the cavity resonances without influence of any instrumental effects and that brodband comb-based systems can be successfully applied to measurements of sub-kHz spectral features.

## Results

**Figure 1:**
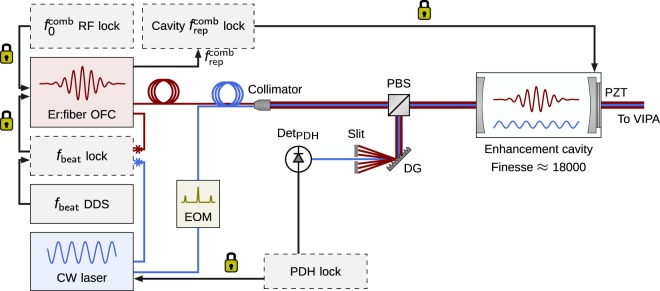
Layout of the experimental setup. The CW laser is tightly locked to the enhancement cavity with the PDH method. The beat note frequency, *f*_beat_, between a single comb tooth and the CW laser is locked with an EOM and a PZT inside the fs laser cavity, narrowing and stabilizing the OFC lines relative to the cavity modes. The *f*_beat_ DDS frequency controls the relative detuning between the OFC and the cavity. The ${f}_{0}^{{\rm{comb}}}$ frequency is locked to an Rb frequency standard. The cavity length and the *f*_rep_ frequency are stabilized by a slow feedback to the cavity PZT. After interacting with the gas sample in the cavity, the combined comb-CW laser beam is coupled into a polarization-maintaining fiber and transferred to the VIPA spectrometer for comb-resolved detection. EOM, electroopic modulator; PBS, polarizing beam splitter; PZT, piezoelectric transducer; Det_PDH_, photodetector for the PDH lock; DG, diffraction grating.

The layout of the experimental setup is shown in Fig. 1. The optical frequency comb (OFC) spanning 1.52–1.59 μm wavelength range is generated by an Er:fiber laser (Menlo FC1500) emitting pulses with the repetition rate *f*_rep_ of 250 MHz. The CW laser (RIO Planex, Redfern Integrated Optics, Santa Clara, CA, USA) is an ECDL laser with emission wavelength centered at 1.564 μm and kHz-level free-running line width. The CW laser light is split and one branch is combined with the OFC light in a fiber coupler to produce a beat note. The remaining CW laser light is modulated at 10 MHz to obtain sidebands for the Pound-Drever-Hall (PDH) locking scheme^31^. The EOM-modulated CW laser is then combined in a fiber coupler with a part of the OFC light which is amplified and optionally Raman-shifted. The output of the fiber coupler is collimated and the mode-matched free-space beam is directed to the enhancement cavity.

The cavity consists of two high-finesse, low-dispersion mirrors (Layertec GmbH, Mellingen, Germany) with reflection coefficient *R* ≈ 0.9998, separated by distance *L* ≈ 0.562 m. This translates to full-width at half-maximum (FWHM) of the cavity resonances of 14.8 kHz and free spectral range (FSR) of 266.6 MHz. One of the mirrors is mounted on a slow piezoelectric transducer (PZT) with 10 µm travel range. In the current realization the cavity is not temperature-stabilized and is susceptible to a drift of 1 K during the day. The ratio of the cavity FSR and the OFC line spacing is a rational fraction, 16/15, which allows only every 16th comb mode to be transmitted^6,32^ and produces a new OFC behind the cavity with the *f*_rep_ of 4 GHz. The cavity output is transferred to a VIPA spectrometer with a polarization-maintaining fiber^19,22,33,34^. The spectrometer consists of a high-finesse etalon, having 600 MHz resolution and free spectral range of 52 GHz, and a diffraction grating with 30 GHz resolution. The combined effect of these elements on the comb beam is to produce a 2D diffraction pattern which is imaged on an InGaAs photodetector array (Xeva-1.7-320, Xenics nv, Leuven, Belgium).

The CW laser is tightly locked to the peak of a cavity resonance with the Pound-Drever-Hall method, which significantly narrows the width of the laser line. The nearest OFC tooth is beat with the CW laser and the beat note frequency *f*_beat_ is mixed with a reference frequency signal generated by a DDS. The DDS frequency and all other radiofrequency (rf) signals are referenced to an Rb frequency standard (FS725, Stanford Research Systems). The mixer output is low-pass-filtered to obtain a phase-difference error signal, which is fed to the EOM and the PZT inside the femtosecond (fs) laser cavity after filtering with a PI controller. The feedback loop phase-locks the comb tooth to the CW laser, acting predominantly on the *f*_rep_ frequency of the OFC^35^. Consequently, any changes in positions of cavity modes are translated into *f*_rep_ changes, which causes the whole OFC to follow the cavity mode structure. The offset frequency ${f}_{0}^{{\rm{comb}}}$ remains as a free parameter, which is set to a value ensuring OFC transmission in a broad spectral range, and is stabilized to an rf reference. The cavity length in turn is stabilized by feeding a signal proportional to the phase difference between the *f*_rep_ and ${f}_{{\rm{rep}}}^{{\rm{ref}}}$ frequency reference to the PZT in the optical enhancement cavity. Stabilizing cavity mode frequencies to an Rb frequency standard establishes absolute frequency axis of recorded spectra and makes this technique suitable for high-accuracy spectroscopy.

### Frequency noise and stability

**Figure 2:**
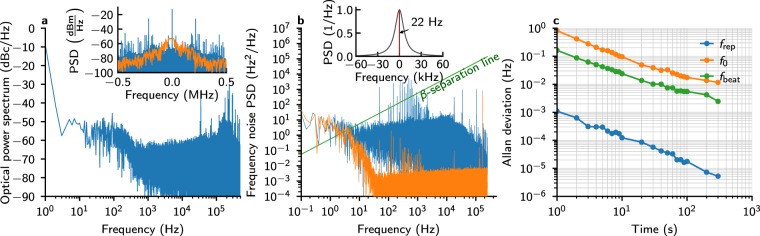
Frequency stability of the system. (**a**) Single-sideband optical power spectrum of the locked OFC-CW laser beat note. Inset: beat note between the PDH-locked CW laser and either the free-running OFC (orange) or the phase-locked OFC (blue). The free-running OFC beat was acquired with RBW of 10 kHz and the phase-locked OFC with RBW of 1 Hz. The fraction of power in the coherent peak is 97%. (**b**) Single-sideband frequency noise of the OFC relative to the enhancement cavity (blue) and the noise floor of the measurement (orange). Inset: the reconstructed lineshape of the OFC (red) as compared to a cavity mode (gray). (**c**) Allan deviation of the frequencies determining the absolute frequency stability of the system.

To verify operation and discover limitations of the technique, we characterized the relative frequency noise and stability of the system components. The relative frequency noise of the unlocked cavity and the free-runing OFC was estimated from the power spectrum of the beat note between the PDH-locked CW laser and an OFC line, shown as an orange trace in the inset of Fig. 2a. The main contributions to the noise are the acoustic noise, causing cavity jitter which broadens the beat note to 80 kHz 3-dB full width at 5.3 ms sweep time, and the free-running comb linewidth. Initiating the *f*_beat_ lock pushes 97% of the beat note power to the coherent peak. To quantify the relative OFC-cavity frequency noise when the system is fully locked, and provide an upper limit for the relative CW laser-cavity linewidth, we have implemented a scheme similar to the one described in ref.^36^. In this scheme OFC lines are detuned from cavity modes to fall on the side of the cavity fringes and their slope is used as a frequency discriminant to measure the relative frequency noise. The OFC was locked to the cavity such that the cavity transmission spectrum was centered around the mirrors’ zero-dispersion point and the transmitted spectrum was narrowed with a Czerny-Turner monochromator. The narrowed spectrum spanned a region with 1-nm FWHM in which the detuning between the OFC modes and cavity modes was constant to within 2 kHz, which ensured that a well-defined relation between optical power deviations and frequency deviations existed. The effective cavity mode shape was recorded by tuning the *f*_beat_ frequency and its derivative was calculated to obtain the voltage-to-frequency conversion factor, *D* = 12.5 kHz/V. The beat note frequency was then tuned to obtain 75% of maximum transmission, which corresponds to a region of a cavity resonance with high slope and good linearity. The obtained frequency noise and the noise floor of the measurement are shown in Fig. 2b as a blue and orange curve, respectively. The noise floor is the frequency-noise-equivalent voltage noise of the detector signal measured with the free-running OFC incident upon it. It was calculated by rescaling the detector signal to the voltage level of the comb-cavity frequency noise signal and applying the conversion factor *D*. The RIN of the OFC is the main contribution to the noise floor, with the exception of noise spikes between 40 Hz and 1 kHz and the spike at 20 kHz, which were also present with no light falling upon the detector. From the crossing point of the *β*-line (=8log(2)/*π*^2^*f*)^37^ and the comb-cavity frequency noise PSD, we obtain an approximate FWHM of 5 Hz. For more detailed information on the optical lineshape of the locked OFC, we performed numerical integration of the relation connecting frequency noise PSD with carrier-domain lineshape^38,39^. The procedure was performed with 100 Hz bandwidth and 0.1 Hz resolution to more accurately estimate the linewidth, from which we obtained the FWHM of 22.2 Hz, and with 60 kHz bandwidth and 10 Hz resolution to better investigate the higher-frequency behavior. The optical line shape was found to be close to Lorentzian up to 1 kHz, after which we observed a noise pedestal at the level of 10^−4^. We also present a comparison between a cavity mode spectrum and the comb lineshape in the inset of Fig. 2b, demonstrating that the narrowing of the OFC lines should be sufficient for faithful retrieval of cavity mode shapes.

The FWHM of the narrowed OFC is consistent with linewidths previously obtained for CW external-cavity diode lasers PDH-locked to high-finesse cavities^40^ and confirms that the locking scheme faithfully transfers the relative phase coherence of the CW laser to the OFC. The noise spikes between 20 Hz and 3 kHz, present in OFC-cavity frequency noise, and the noise spikes starting at 200 kHz, present in both OFC-cavity frequency noise spectrum and CW-OFC optical beat note spectrum, were also found in the PDH error signal and in the *f*_beat_ error signal while the CW laser was not locked to a cavity mode. Since the only common elements in each of these cases are the CW laser and its current driver, we attribute the noise to mechanical vibrations of the laser’s cavity and electrical noise of the current driver.

We have also considered two possible additional broadening effects specific to our locking scheme and by a conservative estimation they do not account for more than 115 mHz of the linewidth. The first one occurs because the ${f}_{0}^{{\rm{comb}}}$ frequency is locked to an rf reference and not directly to the cavity, and the changes in the offset frequency of the cavity caused by its length changes are not followed by the OFC; this amounts to a 109-mHz broadening. The second one occurs only when the offset frequencies of the OFC and the cavity are unequal, leading to a slight mismatch between the *f*_rep_ and FSR frequencies, and scales with the distance from the CW locking point; this amounts to a 6-mHz broadening for a comb line 100 cm^−1^ away from the locking point.

The absolute frequency stability of the system is determined by the stability of the enhancement cavity. In the long term the frequencies of cavity modes drift as temperature changes. This drift is removed by stabilizing *f*_rep_ with the cavity PZT lock as shown by the Allan deviation of *f*_rep_ in Fig. 2c. The Figure also shows the stability of the two other frequencies which jointly with *f*_rep_ determine absolute positions of the OFC modes, proving the technique’s suitability for absolute frequency metrology. The cavity PZT lock performance is limited by the noise of the loop’s phase-discriminant. We estimate the noise floor to be 3 × 10^−7^ Hz^2^/Hz at 1 Hz, with the noise scaling as frequency squared, which translates to 1.5 × 10^4^ Hz^2^/Hz at 1 Hz in the optical domain. The gain and the bandwidth, less than 5 Hz, is kept low enough to not imprint the rf noise onto the carrier-domain lineshape and as a consequence the short-term absolute frequency stability is determined by the natural stability of the optical cavity. Conversely, the operation of the loop has a negligible effect on the OFC-cavity frequency noise at the frequencies shown in Fig. 2b.

### Spectroscopy

To verify the accuracy of mode width and mode position measurements and check for possible systematic effects, we applied the CMDS and CMWS techniques to measure dispersion and absorption lineshapes of rovibrational transitions in in the second overtone band of CO. The gas sample contained 0.1% of CO in N_2_ under 714 Torr pressure at 21.5 °C temperature. A single simultaneous measurement of 412 cavity modes in 55 cm^−1^ span with 4-GHz spectral element with minimal averaging time is 1 s. The bandwidth of the VIPA spectrometer determined by physical dimensions of the camera is equal to 80 cm^−1^, corresponding to 600 cavity modes, but the bandwidth of a single comb-resolved tranmission spectrum was limited to approx. 50 cm^−1^ by dispersion of the cavity mirrors. The measurement of CO-N_2_ spectra consisted of scanning the cavity modes over OFC lines in 40 steps separated by Δ*f*_beat_ = 3 kHz. We elected to acquire at each step 50 signal and dark frames with 10 ms integration time and 60 ms acquisition time for preliminary noise averaging, which resulted in a 4-minute scan. Here, acquisition time is the real measurement time of a single camera frame, which consists of integration time adjustable down to 1 μs, in which photoinduced charge is collected in individual pixels, and a 50 ms dead time required for charge readout, ADC conversion, data transfer and processing. The dead time can be reduced to 10 ms with data processing step optimized or live processing disabled. Each 4-minute scan was repeated 8 times to establish noise averaging properties of the system and to minimize the influence of potential laser intensity drift or variations of amplified OFC spectrum on retrieved mode shapes. The whole 32-minute procedure was then repeated at 3 additional *f*_rep_ values and the resulting spectra were interleaved to reduce the spacing of spectral elements from 4 GHz to 1 GHz. The total measurement time was 128 minutes and the whole procedure was automated, except the changes of *f*_rep_.

It is worth noting that because in CMWS and CMDS techniques the cavity modes are scanned over the comb lines, the mirror-dispersion bandwidth limit is not a hard limit, but it makes it necessary to scan a larger span of *f*_beat_ frequencies to cover a larger spectral bandwidth, leading to longer scan times. Since the reported measurements consist of multiple cavity-enhanced comb-resolved absorption measurements, which are then interleaved and processed to determine the cavity mode widths and mode positions, the experimental setup can be also utilized for regular comb-resolved cavity-enhanced absorption spectroscopy with only a change in the measurement procedure. In this case, instead of scanning over cavity modes, single signal, reference and dark frames are acquired, resulting in minimal measurement time of 30 ms.

**Figure 3:**
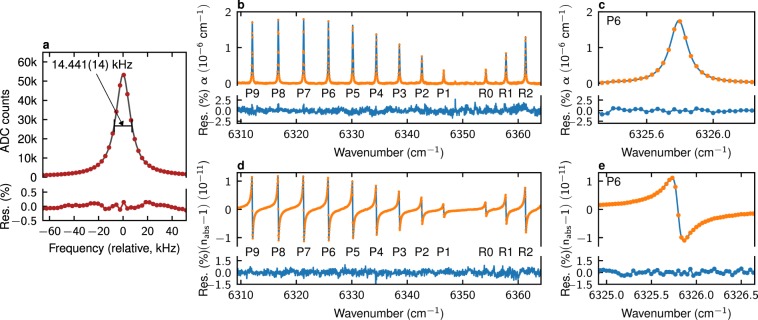
Cavity mode-width and mode-dispersion spectra of a CO-N_2_ sample. (**a**) The shape of a cavity mode unbroadened by gas absorption at 6347.5 cm^−1^ and its Lorentzian fit. The 1*σ* fit uncertainties of its halfwidth and position are 7 Hz and 4 Hz, respectively. (**b**–**e**) Broadband dispersion and absorption spectra of CO mixed with N_2_. The orange circles are the experimental data, the blue lines are the results of simultaneous fits of the Voigt profile to all lines. The residuals are shown in percents of peak absorption or peak-to-peak dispersion for absorption and dispersion measurements, respectively. (**c**,**e**) Zoom-ins of the global fits and experimental data on the P6 line.

Since for our cavity mirrors with *R* = 0.9998 non-resonant cavity transmission is below 10^−7^ of incident power and thus outside of the dynamic range of our detection system, measured cavity modes shouldn’t exhibit any background, *i.e*. they should be described by a purely Lorentzian shape and characterized only by their amplitude, position and width. To check for spurious background, which would point to improper operation of the system, we have performed fits of all measured modes without any baseline function and with a quadratic baseline. Comparing these two cases did not reveal any differences, validating our measurement. Further confirmation was found by comparing the cavity mode width obtained by measuring cavity ringdowns after shutting off the CW laser beam — 13924 ± 144 Hz, with the cavity mode width obtained by extrapolating comb measurements to the wavelength of the CW laser — 13808 Hz. An example cavity mode with the FWHM of 14.441 kHz and its Lorentzian fit with 1*σ* uncertainties of position and FWHM equal to 14 Hz and 4 Hz, respectively, are shown in Fig. 3a.

These results and the foregoing analysis of frequency noise and stability allows us to establish the resolution, precision and accuracy of the spectrometer in regards to cavity mode measurements. The frequency resolution is defined by the comb-cavity FWHM equal to 22.2 Hz. In contrast to the CRDS technique, this resolution is not compromised by measuring transient ring-down events whose spectral width is defined by the cavity mode widths. The OFC lines resonant with the cavity preserve their measured width of 22.2 Hz and in principle this is the frequency resolution limit for any absorption feature sampled by the comb lines. The precision is determined by cavity mode fit uncertainties and for mode positions is equal to 5 Hz at maximum averaging time of 33 minutes, corresponding to fractional precision of 2.7 · 10^−14^ and noise averaging rate of 198 Hzs^1/2^. For mode widths, we obtain precision of 8.7 Hz at the maximum averaging time, corresponding to fractional precision of 1.2 · 10^−3^ and averaging rate of 345 Hz s^1/2^. The frequency standard used is accurate to within 2 · 10^−11^ at 1 s and 2 · 10^−12^ at 100 s. This stability is directly transferred to relative OFC-cavity stability by referencing the DDS generating *f*_beat_ reference frequency and does not limit the mode width measurements. In the case of cavity mode positions, the absolute frequency accuracy is limited by the stability of *f*_rep_. The fourth harmonic of *f*_rep_ is stabilized to the frequency standard, which results in absolute frequency accuracy of cavity mode positions of 22.2 Hz at maximum averaging time, corresponding to fractional accuracy of 1.2 · 10^−13^ and averaging rate of 0.95 kHz s^1/2^.

**Figure 4:**
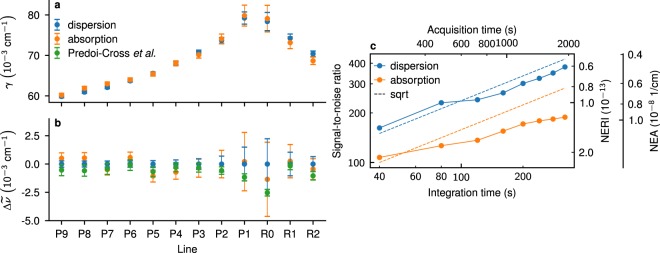
Line widths and line positions in absorption and dispersion spectra. Noise averaging. (**a**) Fitted halfwidths with 2*σ* fit uncertainties as error bars. (**b**) Differences in line positions between dispersion, absorption and reference measurements^42^. The zero level corresponds to line positions from dispersion measurements. The error bars are 2*σ* fit uncertainties for dispersion and absorption measurements, and the combined uncertainties of HITRAN transition frequencies and Predoi-Cross pressure shift coefficients for reference values. (**c**) Signal-to-noise ratios and the corresponding noise-equivalent absorption (NEA) and noise-equivalent refractive index (NERI) values for absorption and dispersion spectra, as a function of integration time and acquisition time of the whole cavity mode.

The fitted mode widths and positions were recalculated to, respectively, effective loss per unit length^29^ and refractive index^17^. In both cases the whole spectrum was fitted simultaneously to a sum of Voigt profiles (VP). Doppler widths and intensities of each line were fixed to constant values based on, respectively, known temperature of the sample and the HITRAN database^41^ line intensities. The fitted concentration of the sample was shared by all lines in the model, thus the parameters specific to individual lines were limited only to their widths and positions, whose initial values were equal to the HITRAN database parameters. For dispersion spectra the baseline was described by a fourth-order polynomial to model the combined dispersion of the pair of cavity mirrors and N_2_ molecule. Since the spectral line shapes are sharp relative to the baseline — the slope on the line is approx. 54 kHz/cm^−1^, while the slope of the baseline is at most 2 kHz/cm^−1^ — and they are well separated from each other, it was sufficient to simultaneously fit the molecular line shapes and the baseline. For measurements of arbitrarily broad spectral features a separate empty-cavity CMDS measurement, performed once for a given pair of mirrors, would be warranted. Additionally, to each *f*_rep_ value a separate set of two sine functions was fitted to account for drifting etalons. For absorption spectra the baseline was modeled by a linear function to account for wavelength-dependent reflection coefficient of the cavity and, similarly to dispersion spectra, a set of two sine functions per each *f*_rep_ value. The fits of both cavity modes and molecular line shapes were performed with the Levenberg-Marquardt algorithm. The resulting absorption (b, c) and dispersion (d, e) fits and experimental spectra are shown in Fig. 3. The fitted absorption and dispersion linewidths and line positions, shown in Fig. 4a,b, were found to be consistent with each other within 2*σ* fit uncertainties. Additionally, the positions were compared with the most accurate previous measurements of N_2_-shifted CO line positions by combining HITRAN transition frequencies and pressure shifts from ref.^42^ (see Table 1). They were also found to be consistent with our measurements within 2*σ* fit uncertainties.

**Table 1:** Positions of the second overtone rovibrational lines of CO in N_2_ at 714 Torr pressure, 21.5 °C temperature.

Wavenumber (cm^−1^)					
Line	Dispersion	Absorption	Diff. (10^−4^)	Predoi-Cross *et al*.	Diff. (10^−4^)
P9	6312.06436 (28)	6312.0638 (5)	5.1	6312.0649 (5)	−5.3
P8	6316.74425 (26)	6316.7437 (5)	5.4	6316.7448 (5)	−5.9
P7	6321.32048 (26)	6321.3210 (5)	−5.0	6321.3209 (5)	−4.3
P6	6325.79304 (28)	6325.7925 (5)	5.8	6325.7931 (5)	−1.0
P5	6330.16057 (31)	6330.1616 (5)	−10.6	6330.1612 (4)	−6.7
P4	6334.4251 (4)	6334.4258 (6)	−7.1	6334.4254 (4)	−2.8
P3	6338.5844 (5)	6338.5848 (8)	−3.6	6338.5848 (4)	−3.2
P2	6342.6396 (7)	6342.6396 (12)	0.0	6342.64013 (33)	−5.8
P1	6346.5893 (15)	6346.5890 (26)	2.1	6346.59042 (31)	−11.6
R0	6354.1732 (22)	6354.1746 (33)	−13.5	6354.17574 (29)	−25.3
R1	6357.8098 (10)	6357.8096 (15)	2.5	6357.80998 (33)	−1.7
R2	6361.3380 (7)	6361.3384 (9)	−4.5	6361.3390 (4)	−10.4

The uncertainties of dispersion and absorption measurements in the parentheses are 2*σ* fit uncertainties. The uncertainties of reference line positions are the combined uncertainties of pressure shifts from ref.^42^ and transition frequencies from the HITRAN database. *Diff*. columns contain differences in line positions between dispersion and absorption measurements, and dispersion and reference values.

The accuracy of the molecular line parameters is not limited by the resolution of the spectrometer, which is determined by the OFC linewidth equal to 22.2 Hz. The precision and accuracy of the measurements is dominated by the signal-to-noise ratio (SNR), compared to which the contributions from cavity mode position uncertainties and frequency reference stability is negligible. The chief advantage of the broadband mode-dispersion technique, which cannot be implemented in conventional Fourier-transform spectrometers based on thermal light sources, is its dependence only on frequency measurements. This links the measurements directly to the SI unit and makes them less susceptible to systematic errors. The measured line shapes and the parameters derived from them fully confirm the accuracy of the retrieved cavity mode widths and positions and the absence of systematic effects.

We estimate the SNR in the spectra by ratio of the peak absorption or the peak-to-peak dispersion to the standard deviation of global fit residuals. The increase in SNR and sensitivity with averaging time is shown in Fig. 4c, as a function of camera integration or acquisition time needed to scan over the whole cavity mode and record bright and dark frames. Allowing for uncertainty in noise estimation, the dispersion curve follows square-root law, whereas absorption spectra are characterized by lower rate of noise averaging. This is possibly caused by residual camera nonlinearity or dependence of fm-to-am noise conversion on detuning between comb teeth and cavity modes, both of which could be responsible for the faint “w” shape present in cavity mode residuals (see Fig. 3a). Since the “w” shape is symmetric, these effects would not affect estimation of cavity mode positions and dispersion spectra. In fact, it is worth noting that any kind of systematic effect which affects the cavity mode shapes in a symmetric manner will not affect dispersion spectra determined from cavity mode positions. In general, SNR in dispersion spectra is twice as large as in absorption spectra. This is consistent with the ratio of relative fit uncertainties of mode widths and positions being also close to two. Both of these effects affecting absorption but not dispersion measurements gives evidence for the claim from CW laser-based measurements that dispersion measurements have an intrinsic advantage over absorption measurements^30^. Our system is not limited by the fundamental noise and we expect the noise averaging rate to improve when acoustic noise isolation is implemented by reducing fm-to-am noise. Alternatively, a locking scheme analogous to *f*_beat_ lock, but acting on the fs laser pump current could be used to directly stabilize the *f*_0_ frequency of the OFC to cavity mode frequencies.

## Discussion

We have presented a broadband spectroscopic system capable of measuring 14-kHz wide cavity resonances with Hz-level precision, demonstrating the highest spectral resolution and frequency precision obtained with a broadband spectroscopic system. This proves that the resolution limits often plaguing incoherent light-based spectrometers can be avoided in comb-based spectroscopic systems and that the inherent precision and accuracy of stabilized OFCs can be fully utilized in broadband spectroscopic measurements. Combined with the high acquisition rate, such spectrometers appear as the proper tool for investigation of narrowest spectral features, for example narrow resonances in cold atoms and molecules or chemical kinetics studies. If single-spectrum acquisition time is of critical importance for a particular application, the system can be used for regular comb-resolved cavity-enhanced absorption measurements. The introduced locking scheme provides a way to arbitrarily detune the OFC relative to the cavity with low relative frequency noise and self-reference the frequency axis of the acquired spectra. The component simple low-bandwidth loop ensures absolute frequency stability and bypasses the necessity of technically challenging passive cavity stabilization. The overall performance of the system was verified by measurements of spectra of a high-pressure CO-N_2_ sample, also demonstrating the utility of this technique for high-resolution spectroscopy and lineshape analysis with longer averaging times. The measurements can be fully automated and the scheme adjusted to obtain spectral bandwidth and comb mode spacing appropriate for a given application. Although not shown here, it would be straightforward to apply the system to a broadband characterization of high-finesse cavity mirrors, both their reflection coefficient and dispersion^17,43,44^, or broadband refractive index measurements^45^. Taking into account the possibility of efficient nonlinear frequency conversion^46–49^, the presented experimental scheme can be relatively easily transferred to other spectral ranges, including the molecular fingerprint region in the mid-infrared part of electromagnetic spectrum.

## Materials and Methods

### VIPA spectrometer

**Figure 5:**
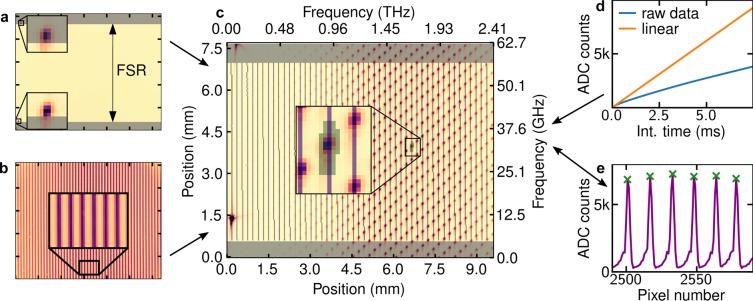
VIPA spectrometer data analysis scheme. (**a**) A CW laser imaged by the spectrometer delimits the region of photodetector array corresponding to one FSR of the VIPA etalon. (**b**) Unresolved OFC spectrum in a form of vertical stripes provides a guide for locating single comb teeth in subsequent measurements. (**c**,**e**) Pixel intensities along vertical stripes are collected and the locations of maxima (green crosses) are used to locate and integrate teeth intensities in comb-resolved measurements (green shaded area). (**d**) The fitted nonlinearity correction is used to calibrate raw data frames.

The VIPA spectrometer allows for accurate determination of resolved comb modes’ intensities provided a proper procedure is followed. The photodetector array used in the experiment exhibits highly sublinear dependence of analog/digital converter (ADC) counts on incident power (see Fig. 5d). This effect led to significant deviations of recorded cavity mode shapes from the Lorentzian shape and significant overestimation of cavity mode widths, thus it was necessary to calibrate the response of individual pixels over the whole ADC range. We recored a set of images at uniform illumination of the photodetector array for a range of integration times and fitted a zero-intercept linear function to dependence of ADC counts on integration time. For each pixel the ratio between the linear function and the measured counts provided a calibration curve, which was used in subsequent measurements. We have verified that periodic recalibration is unnecessary by comparing measurements separated by six-months time period. We have found the camera dark current to drift over timescale of tens of seconds. To mitigate this effect, during measurement acquisition of bright signal frames is interleaved with acquistion of dark frames and the procedure is automated with an Arduino-controlled mechanical shutter. Additionaly, a part of the diffraction pattern was diffusely reflected inside the photodetector head and subsequently reached the array. Since the exact diffraction pattern depends on comb-cavity mode detuning, this resulted in a spurious baseline of recorded cavity mode shapes, which was eliminated by fitting the camera head with a pinhole.

After recording a set of bright and dark frames, subtracting them and calibrating the intensity, single comb modes can be identified and their intensities can be retrieved. A CW laser is imaged with the VIPA spectrometer and a frame with two bright dots is obtained (see Fig. 5a). The dots correspond to the same frequency diffracted in the first and second interference order of the etalon, and delimit the region in which unambiguous mapping between positions and frequencies is possible. Additional limiting of the region is obtained by imaging a broadband light source, e.g. a frequency comb with *f*_rep_ below the VIPA resolution, which results in a pattern of almost-vertical stripes determined by the combined effect of VIPA and grating dispersion (see Fig. 5b). The stripe pattern is saved and used to analyze frames containing resolved OFC modes. The intensities of the pixels lying on the stripes are collected and positions of third-order intensity maxima are taken to be positions of single comb modes (see Fig. 5d). The intensities of comb modes are obtained by integrating a predetermined pattern of pixels centered at each comb mode peak position and collected in frequency-increasing order (see Fig. 5c). The CW laser, visible on the left edge of Fig. 5c, provides an absolute frequency reference marker, which is used to identify the imaged comb teeth and generate an absolute frequency scale. The comb mode number assignment is performed by first finding the comb mode closest to the CW laser, out of the ones lying on the same stripe; then, the wavelength of the CW laser is determined by coercing the value returned from a measurement by a wavelength meter to the wavelength grid defined by the comb equation, *ν*_CW_ = *j* × *f*_rep_ + *f*_0_ + *f*_beat_; finally, the frequency relations described in the next paragraph are used to identify the comb mode from the first step and all the other imaged comb teeth.

### Vernier scheme

**Figure 6:**
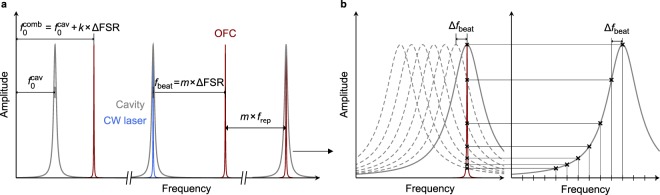
Frequency relations between the OFC, the cavity and the CW laser locked to each other. (**a**) Left: In the Vernier scheme a subset of comb teeth identified by the *k* integer is resonant with the cavity when ${f}_{0}^{{\rm{comb}}}={f}_{0}^{{\rm{cav}}}+k\times {\rm{\Delta }}\mathrm{FSR}$. Middle: If both the OFC and the CW laser are resonant with the cavity, the beat note frequency is equal to *f*_beat_ = *m* × ΔFSR. Right: Based on the CW laser and *f*_beat_ frequencies the closest comb mode resonant with the cavity can be identified. (**b**) Left: Changing the *f*_beat_ frequency shifts the cavity modes and changes intensities of transmitted comb modes. Right: By collecting comb mode intensities at different *f*_beat_ values the shapes of cavity modes are reconstructed.

The Vernier scheme used for filtering the OFC modes introduces ambiguity into the transmitted spectrum. For the Vernier ratio defined by the following equation, *Nf*_rep_ = (*N* − 1)FSR, only every *N*th tooth is transmitted and the OFC spectrum is divided into *N* disjoint subsets, with each of them resonant with the cavity only at a specific detuning between the offset frequencies of the cavity and the OFC. The subsets can be distinguished by an integer, *k*, which is the number of the first comb tooth resonant with the cavity. At zero offset frequency detuning *k* is equal to zero and the next tooth is offset from the closest cavity mode by ΔFSR = FSR − *f*_rep_, so at detuning ${\rm{\Delta }}{f}_{0}={f}_{0}^{{\rm{comb}}}-{f}_{0}^{{\rm{cav}}}={\rm{\Delta }}\mathrm{FSR}$, it will be in resonance. By induction, at detuning Δ*f*_0_ = *k* × ΔFSR the *k*th tooth will be transmitted, with *k* spanning the range from 0 to *N* − 1 and the subsets indexed by *k* covering the whole comb spectrum. An implication of these relations important for the presented locking scheme is that when the OFC and the cavity are in resonance the frequency difference between a comb tooth and a cavity mode can only ever be equal to a multiple of ΔFSR, thus the allowed *f*_beat_ values are also restricted in such way, since the CW laser is fixed to the peak of a cavity mode. The frequency of the CW laser can be effectively treated as a new origin of the frequency axis with ${\rm{\Delta }}{f}_{0}^{{\rm{eff}}}={f}_{{\rm{beat}}}=m\times {\rm{\Delta }}\mathrm{FSR}$, and the value of *m* providing the number of the nearest transmitted higher-frequency comb mode relative to the CW-locked comb mode, which is the information needed for referencing the spectra to the OFC axis. Figure 6a summarizes the most important relations between the OFC, the CW laser and the cavity. As shown in Fig. 6b, the profiles of cavity modes are measured by scanning the *f*_beat_ frequency and recording the transmitted spectra. Since the *f*_rep_ and *f*_0_ frequencies are fixed, it is the cavity modes which are shifted by a Δ*f*_beat_ frequency change of the beat note, each of them by ${\rm{\Delta }}\,{\nu }_{0}^{{\rm{cav}}}=(j/{j}_{{\rm{CW}}}){\rm{\Delta }}{f}_{{\rm{beat}}}$, where *j*_CW_ is the number of the cavity mode to which the CW laser is locked.

## Data Availability

The datasets generated during and/or analysed during the current study are available from the corresponding author on reasonable request.
